# Veterinary Medical Association, State of New Jersey

**Published:** 1895-05

**Authors:** S. Lockwood

**Affiliations:** Secretary


### VETERINARY MEDICAL ASSOCIATION, STATE OF NEW JERSEY.

The annual meeting of the Veterinary Medical Association of New Jersey was held at the State Street House, Trenton, on Thursday, April 11th.

The reports of the Secretary and the Treasurer show the Association to be in a flourishing condition. The officers elected are: *President*, Dr. J. W. Hawk, Newark; *First Vice-President*, Dr. J. Gerth, Newark; *Second Vice-President*, Dr. R. D. Hasbrouck, Passaic; *Secretary*, Dr. S. Lockwood, Woodbridge; *Treasurer*, Dr. B. F. King, Little Silver; *Trustees*, Drs. W. B. E. Miller, L. P. Hurley, R. O. Hasbrouck, W. Runge, and J. C. Dustan.

The number of applications for membership received at this meeting should infuse new life in the older members.

The revision of the constitution and by-laws and other important business consumed so large a part of the day that but one paper was read, entitled "The Necessity of a Thorough Diagnosis for the Successful Treatment of Animals."

A larger number of cases were reported than usual.

The next meeting will be held in Newark in October.

S. LOCKWOOD,
Secretary.

